# The HIF1α/JMY pathway promotes glioblastoma stem-like cell invasiveness after irradiation

**DOI:** 10.1038/s41598-020-75300-5

**Published:** 2020-10-30

**Authors:** Laurent R. Gauthier, Mahasen Saati, Hayet Bensalah-Pigeon, Karim Ben M’Barek, Oscar Gitton-Quent, Romane Bertrand, Didier Busso, Marc-André Mouthon, Ada Collura, Marie-Pierre Junier, Hervé Chneiweiss, José R. Pineda, François D. Boussin

**Affiliations:** 1 Université de Paris and Université Paris-Saclay, Inserm, LRP/iRCM/IBFJ CEA, UMR Stabilité Génétique Cellules Souches et Radiations, 18 route du panorama, 92265 Fontenay-aux-Roses, France; 2 Sorbonne Université, UPMC Univ Paris 06, INSERM, UMRS 938, Equipe Instabilité des Microsatellites et Cancer, Centre de Recherche Saint Antoine, 75012 Paris, France; 3 CNRS UMR8246, Inserm U1130, Neuroscience Paris Seine-IBPS, UPMC, Sorbonne Universités, Paris, France; 4 Present Address: Inserm U861, I-Stem, CECS, UEVE, AFM, Institute for Stem Cell Therapy and Exploration of Monogenic Diseases, 91100 Corbeil-Essonnes, France; 5 Present Address: Achucarro Basque Center for Neuroscience, Sede Building, 48940 Leioa, Vizcaya Spain

**Keywords:** CNS cancer, Cell invasion, Actin, Cancer stem cells

## Abstract

Human glioblastoma (GBM) is the most common primary malignant brain tumor. A minor subpopulation of cancer cells, known as glioma stem-like cells (GSCs), are thought to play a major role in tumor relapse due to their stem cell-like properties, their high resistance to conventional treatments and their high invasion capacity. We show that ionizing radiation specifically enhances the motility and invasiveness of human GSCs through the stabilization and nuclear accumulation of the hypoxia-inducible factor 1α (HIF1α), which in turn transcriptionally activates the Junction-mediating and regulatory protein (JMY). Finally, JMY accumulates in the cytoplasm where it stimulates GSC migration via its actin nucleation-promoting activity. Targeting JMY could thus open the way to the development of new therapeutic strategies to improve the efficacy of radiotherapy and prevent glioma recurrence.

## Introduction

Glioblastoma (GBM) is the most common and aggressive type of primary brain tumor^1^. The standard GBM treatment includes surgical resection with adjuvant radiotherapy and chemotherapy^2^. The radiotherapy treatment usually consists of five consecutive sessions of 2 Gy rate per week for six weeks, the total treatment being 60 Gy^2^. However, the majority of patients relapse and only 3 to 5% survive more than three years following treatment^3^. The highly infiltrative nature of GBM is thought to contribute to tumor relapse^4^. Moreover, ionizing radiation (1–20 Gy) has been shown to promote the migration and invasion of human glioblastoma cell lines in vitro^5–17^ suggesting that this effect may allow some cancer cells to move away from the irradiated area, thereby reducing their radiation exposure at next irradiation sessions and overall the treatment efficacy.

Multiple studies have shown that GBM displays intratumoral heterogeneity with a hierarchical cellular organization stemming from a minor subpopulation known as glioma stem-like cells (GSCs), which are able to generate intracerebral tumor in immunodeficient mice^18,19^ and share some properties with normal neural stem cells, including *i)* the expression of specific markers, *ii)* a capacity for self-renewal and *iii)* the ability to give rise to differentiated cells^20–22^. Their stem-like cell potential combined to their high resistance to available cancer treatments and their high invasion capacity^23–25^ suggest that GSCs are involved in GBM relapse following treatment^23,26^.

Here, we demonstrate that sublethal doses ionizing radiation specifically promotes the migration and invasiveness of human GSC lines using in vitro and in vivo assays. We show that radiation-induced migration/invasion occurs through the stabilization and nuclear accumulation of the transcription factor hypoxia-inducible factor 1 alpha (HIF1α), which drives the transcription of Junction-mediating and regulatory protein (JMY)^27^ that stimulates GSC migration through its actin nucleation-promoting activity.

## Results

### γ-radiation increases the migration velocity and invasive capacity of human GSCs

We used time-lapse videomicroscopy to characterize the motility patterns of two human GSC lines: TG1N and TG16, which were obtained from patients with high-grade gliomas^28,29^. Since then they were systematically cultured as tumorospheres in defined stem cell culture conditions, allowing them to keep their GSC properties including their capacity to generate intracerebral tumors in immunodeficient mice (Supplementary Fig. S1A).

Twenty-four hours after plating on laminin substrate, TG1N and TG16 cells adopted a bipolar and elongated shape (Supplementary Fig. 1B) and displayed high motility (mean velocities of 26.3 ± 0.6 µm/h and 25.7 ± 1.1 µm/h, respectively) without a predefined direction (Supplementary Fig. S1C, Supplementary Movies S1 and S2), consistently with random motility pattern with high velocity previously reported for other GSC lines^30^.

We then determined the effects of different ionizing radiation doses ranging from 0 to 3 Gy on the motility pattern of TG1N and TG16 cells. In agreement with the well-known radiation-resistance of GSCs^23,29^, quantification of activated caspase-3 and -7 in irradiated cultures by ELISA revealed minimal increases in apoptosis at 24 h post-irradiation, even at the highest dose (Supplementary Table S1). This was further confirmed by using IncuCyte Cytox Reagent to assess cell death by videomicroscopy at different times after irradiation (Supplementary Table S2). Flow cytometric analysis with propidium iodide DNA staining at 24 h post-irradiation revealed no effect of 0.5 Gy irradiation on the cell cycle of TG1N and TG16 and only a low G2/M accumulation after 3 Gy in cultures of both cell lines (Supplementary Table S3). Similarly, the colony formation assay revealed that only the dose of 3 Gy significantly impairs clonogenicity of both TG1N and TG16 cells (Supplementary Fig. S2).

**Figure 1:**
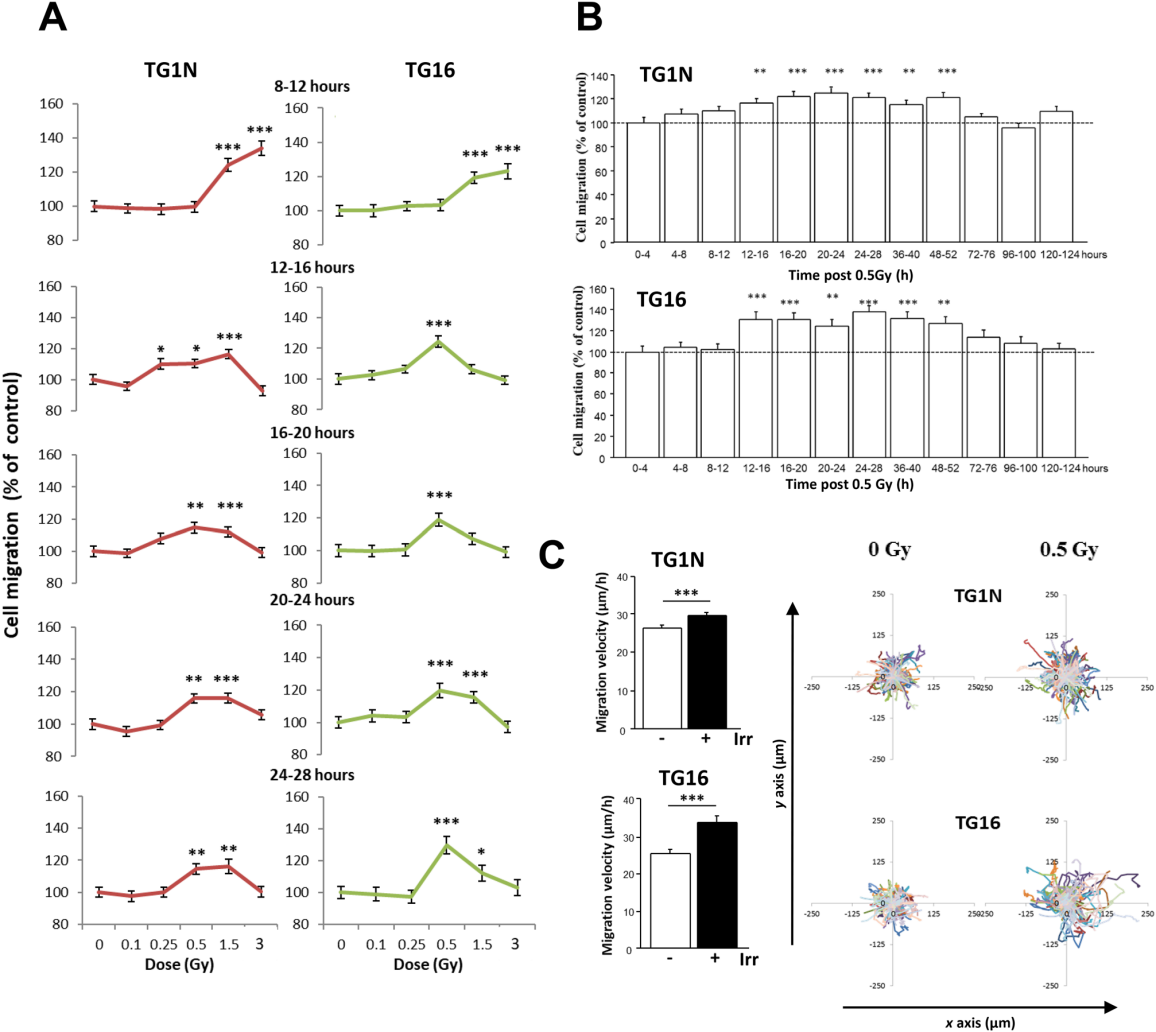
γ-irradiation stimulates the migration of GSCs. (**A**) Dose-dependent effects of radiation on the migration of TG1N and TG16 GSCs. Cell motility was monitored by videomicroscopy for successive 4-h periods from 8 to 28 h post-irradiation. Graphs show the effects of radiation on cell migration expressed as percentages of the respective unirradiated controls for the indicated period of time after irradiation. Percentages of mean migration velocity were calculated from at least 120 cells (TG1N) and 80 cells (TG16) from two independent experiments (*p < 0.05, **p < 0.01 and ***p < 0.001). (**B**) Effects of 0.5 Gy irradiation on the migration of human TG1N (top) and TG16 cells (bottom). GSCs were irradiated 24 h after plating. Histograms show the effects of radiation on cell migration expressed as percentages of the respective unirradiated controls during the indicated periods of time after irradiation. At least 80 cells (TG1N) and 90 cells (TG16) were tracked every 10 min over the course of 124 h for each period of 4 h. Data were compiled from three independent experiments. (**p < 0.01 and ***p < 0.001). (**C**) Mean migration velocity and cumulative traces of control and irradiated (0.5 Gy) TG1N, and TG16 cells from 24 to 28 h after 0.5 Gy irradiation. Histograms on the left show the mean velocities of irradiated cells and non-irradiated controls (calculated from at least 75 cells per condition; ***p < 0.001). Graphs on the right generated using DiPer program^58^ show the color traces representing the tracking of 50 individual irradiated cells and non-irradiated controls. Similar results have been reproduced in at least three other independent experiments.

GSC migration velocity was measured over periods of 4 h ranging from 8–28 h post-irradiation. We showed dose-dependent increases of migration velocity of irradiated cells as compared to that of unirradiated controls, which remained stable during this period of time (Fig. 1A). No increase was detected after 0.1 Gy, whereas the highest increase was observed at 8–12 h after 3 Gy irradiation (1.34- and 1.23-fold increases for TG1N and TG16, respectively, ***p < 0.001; Fig. 1A). Migration velocity decreased thereafter at the highest dose probably due to the cell cycle alterations reported above (Supplementary Table S2 and S3). By contrast, we showed that 0.5 Gy induced a persistent increase in the migration velocity of the two cell lines, which remained detectable up to 52 h post-irradiation (Fig. 1B).

Irradiated (0.5 Gy) GSCs significantly explored a wider territory than unirradiated controls as shown by cumulative traces (Fig. 1C) and mean square displacement (MSD) measurements (Supplementary Fig. S3A,B) of cells tracked from 24 to 28 h or 24 to 26 h post-irradiation respectively. This occurred without any change in directional persistence estimated either by the end-point method (defined as the ratio of the distance between two points by the actual trajectory; Supplementary Fig. S3C,D) or over time (every 10 min, Supplementary Fig. S3E,F).

**Figure 2:**
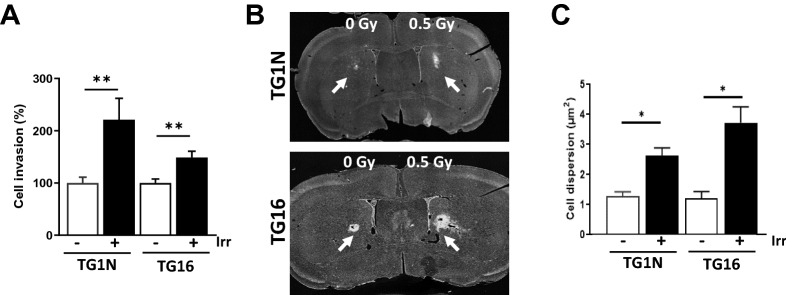
Irradiation increases the invasiveness of GSCs in vitro and in vivo. (**A**) Matrigel invasion chambers were used to measure invasiveness of TG1N and TG16 cells after 0.5 Gy irradiation. The results are expressed as percentages of non-irradiated controls (n = 5 chambers per group from 3 independent experiments; **p < 0.01). (**B**) Human nestin immunostaining (arrows) of coronal slices of brains of nude mice 48 h after striatal graftings of unirradiated (left) or irradiated (0.5 Gy, right) TG1N cells (upper panel) or TG16 cells (lower panel). (**C**) Quantification of dispersion of unirradiated or irradiated TG1N and TG16 cells in coronal slices of mouse brains Data were compiled from two independent experiments including 3 to 5 brains per condition (*p < 0.05).

We then tested the effect of radiation on GSCs invasiveness using the Matrigel invasion chamber assay. As shown in Fig. 2A, 0.5 Gy significantly increased the invasiveness of both GSC lines (TG1N: 221 ± 41% and TG16: 125 ± 11%).

To further explore in vivo the effects of radiation on GSC invasiveness, irradiated (0.5 Gy) TG1N and TG16 cells were stereotaxically injected into the striatum of adult Nude mice (Fig. 2B). Serial coronal brain slices obtained two days after engraftment revealed that human nestin-positive cells exhibited a greater dispersion in the coronal plane, when cells were irradiated prior to injection compared to unirradiated controls (Fig. 2C).

Altogether, our data showed that sublethal doses of irradiation stimulate both the motility and invasive capacity of human GSCs.

### Radiation-induced migration of GSCs depends on a rapid and transient nuclear accumulation of HIF1α

HIF1α has been shown to play a key role as a transcription factor in hypoxia-induced migration/invasion of several glioblastoma cell lines^9,31–35^. Since HIF1α nuclear accumulation has been previously reported to be induced by ionizing radiation in tumor cells^36^, we investigated whether HIF1α could be involved in the radiation-induced migration/invasion of GSCs.

In normoxia, HIF1α is hydroxylated by prolyl hydroxylase (PHD) leading to its recognition by the von Hippel-Lindau protein and subsequent ubiquitination and targeting to the proteasome for rapid degradation^37^. PHD destabilization under hypoxic conditions allows the accumulation of HIF1α and its translocation to the nucleus^38^, where it forms a heterodimeric transcription factor complex with HIF1ß and binds the promoter regions of target genes^39^.

**Figure 3:**
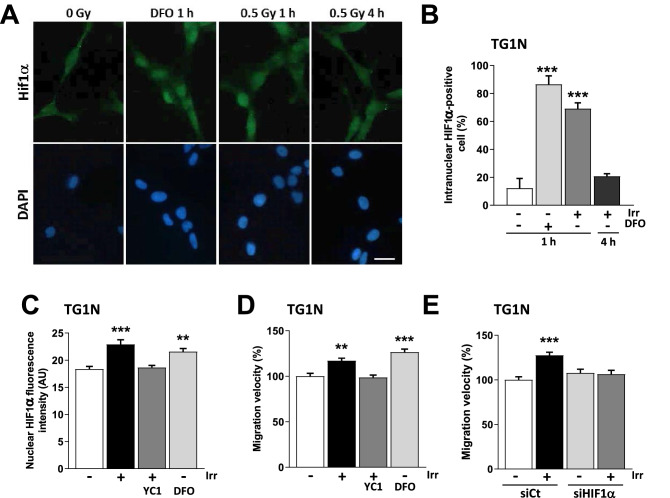
A rapid and transient nuclear accumulation of HIF1α is involved in radiation-induced migration of GSCs. (**A**) Immunostaining of HIF1α in DFO-treated (100 µM, 1 h) or irradiated (0.5 Gy 1 or 4 h PI) TG1N GSCs. Nuclei were counterstained with DAPI. Scale bar: 20 µm. (**B**) Percentage of intranuclear HIF1α-positive cells after DFO treatment or after (0.5 Gy) irradiation (n = 100 cells per condition; ***p < 0.001). (**C**) YC1 (50 µM) treatment 2 h prior to irradiation (0.5 Gy) prevented the radiation-induced nuclear accumulation of HIF1α in TG1N GSCs. One hour after irradiation or DFO treatment (100 µM), nuclear HIF1α fluorescence intensity was determined in at least 50 cells per condition (**p < 0.01 and ***p < 0.001). (**D**) Effects of YC1 or DFO treatments on TG1N GSCs migration velocity 24 h PI. Migration velocity was expressed as percentages of the unirradiated control and calculated from at least 80 cells for each condition (**p < 0.01 and ***p < 0.001). (**E**) HIF1α knockdown prevented the radiation-induced migration of TG1N cells. TG1N GSCs were transfected with a siRNA targeting HIF1α (siHIF1α) or a scramble control (siCt) and irradiated (0.5 Gy) 24 h later. Twenty-four hours after irradiation, migration velocity was determined and expressed as percentage of the unirradiated control. Data were obtained from at least 70 cells per condition (***p < 0.001).

To investigate the role of HIF1α in radiation-induced migration, we treated our cells with Deferoxamin (DFO), an iron chelator known to stabilize HIF1α^40^. As shown in Fig. 3A–C, 100 µM DFO induced the nuclear accumulation of HIF1α in 86 ± 6% of TG1N cells compared to 12 ± 7% of controls (***p < 0.001). Similar data were obtained with TG16 cells (Supplementary Fig. S4D).

Strikingly, we found a similar nuclear accumulation of HIF1α in 70% of irradiated TG1N cells at 1 h post-irradiation, compared to control cells (Fig. 3A,B, ***p < 0.001). This increase remained transient since the percentage of HIF1α-positive cells, as well as nuclear HIF1α intensity returned to control levels at 4 h post-irradiation (Fig. 3A,B and Supplementary Fig. S4A,B). Confirming the radiation-induced activation of the HIF1α pathway, we showed an increased transcriptomic expression of well-known HIF1α target genes in irradiated-GSCs^41^ (Supplementary Fig. S5A).

We next showed that the radiation-induced accumulation of HIF1α did not involve transcriptional regulation, since HIF1α mRNA levels remained unchanged in irradiated cells compared to unirradiated controls, consistently with the determinant role of post-transcriptional modifications in HIF1α accumulation^42^ (Supplementary Fig. S4C). Interestingly, DFO treatment increased cell velocity (Fig. 3D; ***p < 0.001), mimicking the effects of irradiation (Fig. 3D; **p < 0.01). Similar data were obtained with TG16 cells (Supplementary Fig. S4E).

To further investigate the importance of HIF1α on radiation-induced migration, we then used YC1, a nitric oxide-independent activator of soluble guanylyl cyclase described to indirectly block HIF1α expression at the post-transcriptional level^43^. We first checked the inhibition efficiency of 50 µM YC1 on HIF1α expression induced by DFO in our cells and under the culture conditions used for these cells (Supplementary Fig. S5B). To this end, we used HIF1α knockdown TG1N GSCs generated by lentiviral vector transduction of small-hairpin RNAs against HIF1α (shHIF1α), which dramatically decreased the HIF1α mRNA basal levels; Supplementary Fig. S5C) and shCt TG1N GSCs, transduced with a lentiviral vector expressing a small-hairpin directed against irrelevant sequence (shCt). Western blot analysis revealed that DFO treatment induced accumulation of HIF1α in the nuclear fraction of shCt TG1N GSCs after 2 h, whereas it was not detected in the cytoplasmic fraction and in untreated controls (Supplementary Fig. S5B). Finally, YC1 inhibited the effect of DFO treatment on the accumulation of HIF1α in the nuclear fraction similarly as the HIF1α knockdown (Supplementary Fig. S5B), showing that YC1 is an efficient inhibitor of HIF1α.

Further demonstrating the role of the HIF1a pathway in radiation-induced migration, YC1 prevented i) the radiation-induced increase of fluorescence intensity of nuclear HIF1α in TG1N (Fig. 3C) and TG16 (Supplementary Fig. S4D), ii) the radiation-induced transcriptomic expression of HIF1α target genes^41^ (Supplementary Fig. S5A) and iii) the radiation-induced velocity of GSCs (Fig. 3D, Supplementary Fig. S4E).

Finally, we treated TG1N and TG16 cells with specific siRNAs that decreased HIF1α mRNA expression by 88–93% (Fig. S4F). As shown in Fig. 3E and S4G, HIF1α knockdown in TG1N and TG16 cells inhibited radiation-induced migration compared to control siRNA (siCt)-transfected cells. Altogether, these data strongly demonstrate the key role for HIF1α in the radiation-induced GSC migration.

### Stimulation of the Hif1α/JMY pathway increases radiation-induced GSC migration

Nuclear HIF1α is known to bind to hypoxia response elements (HRE) present in the promoters of a large number of genes^44^; these genes encode proteins critical for many important cellular processes, including migration^45^. Junction-mediating and regulatory protein (JMY) is one of the genes whose transcription is driven by HIF1α under hypoxic conditions^46^. JMY has also been reported to enhance cell motility and invasion via its ability to induce actin nucleation^47,48^.

**Figure 4:**
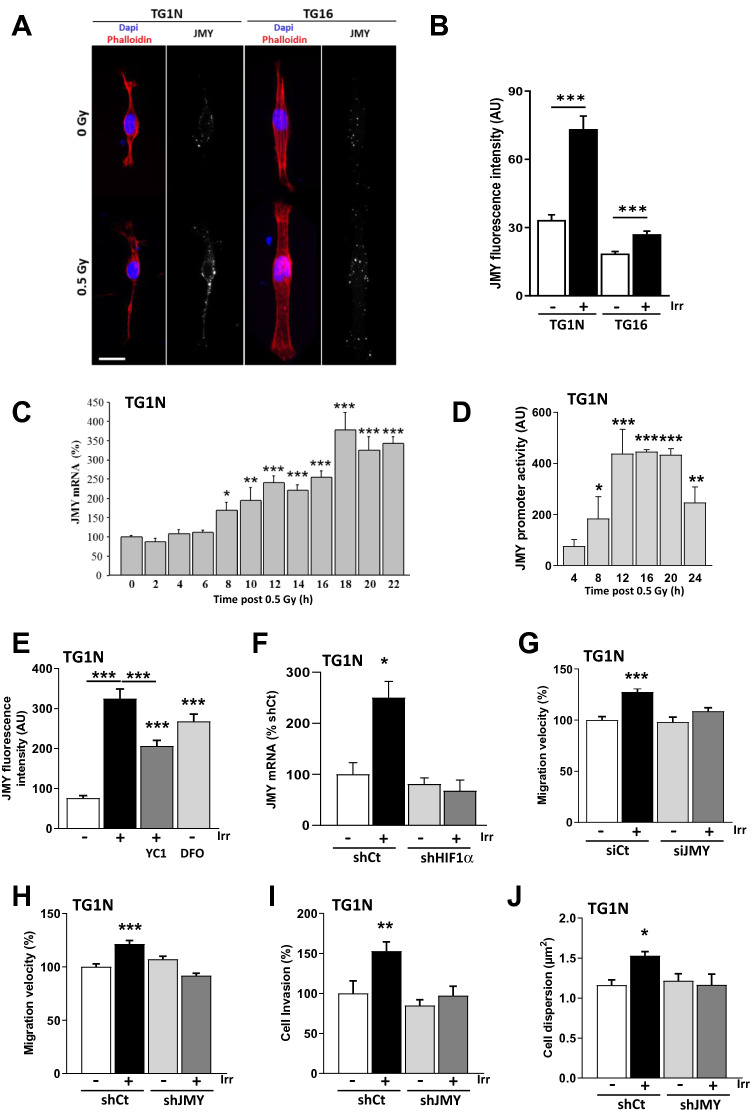
The HIF1α/JMY pathway is involved in radiation-induced migration of GSCs. (**A**) JMY immunostaining (white) of GSCs 24 h after 0 Gy or 0.5 Gy irradiation. F-actin filament networks are stained with fluorescent-phalloidin (red). Nuclei were counterstained with DAPI (blue). Scale bar: 20 µm. (**B**) Quantification of JMY fluorescence intensity. At least 20 cells were scored per condition. (***p < 0.001). (**C**) Quantification of JMY mRNA levels by RT-qPCR in TG1N cells. Experiments were performed in triplicate (*p < 0.05, **p < 0.01 and ***p < 0.001). (**D**) JMY promoter activity in TG1N cells was estimated by luciferase reporter assay at different times following irradiation (0.5 Gy). Data obtained from quadruplicates (*p < 0.05, **p < 0.01 and ***p < 0.001). (**E**) TG1N cells were treated with 50 µM YC1 one hour before irradiation (0.5 Gy). Immunostainings were performed 24 h after irradiation or after treatment with 100 µM DFO. JMY-fluorescence intensity was measured (n = 30 cells per condition; ***p < 0.001). (**F**) Quantification of JMY mRNA levels by RT-qPCR in control TG1N cells (shCt) and in HIF1α-deficient TG1N cells. Experiments were performed in duplicate (*p < 0.05). (**G**,**H**) Twenty-four hours after irradiation (0.5 Gy), cells transfected with siCt or siJMY (**F**) or transduced with shJMY-TG1N or shCt-TG1N cells (**G**) were tracked every 10 min for 4 h. Data were obtained from at least 65 cells per condition (***p < 0.001). The results are expressed as percentages of unirradiated controls (siCt TG1N cells (**F**) or shCt TG1N cells (**G**)). (**I**) Invasion in Matrigel chambers of ShJMY-TG1N cells and ShCt-TG1N cells after 0.5 Gy radiation. GFP-positive cells present on the lower membrane were numbered. For each condition, results were expressed as percentages of unirradiated ShCt-TGIN cells (n = 3 chambers per group; **p < 0.01). (**J**) Quantification of the dispersion of unirradiated or irradiated ShJMY-TG1N cells and ShCt-TG1N cells in coronal slices of nude mouse brains 48 h after intrastriatal injections (n = 3–5 brains per condition; *p < 0.05).

Immunofluorescence revealed that JMY was significantly up-regulated both in TG1N and TG16 cells 24 h after 0.5 Gy irradiation (Fig. 4A,B; ***p < 0.001). RT-qPCR showed an increase in JMY mRNA levels detectable from 8 h post-irradiation and persisting thereafter (Fig. 4C and Supplementary Fig. S6A). As shown in Supplementary Fig. S7, the activation kinetic of JMY after irradiation is comparable to that of other well-known HIF1α target genes^41^. Moreover, measurement of JMY promoter activity in TG1N cells using luciferase assays confirmed that irradiation induced the activation of the JMY promoter (Fig. 4D).

Strikingly, stabilizing HIF1α levels with DFO increased the expression of JMY, whereas blocking HIF1α with YC1 prevented the irradiation-induced increase in JMY in both TG1N and TG16 cells (Fig. 4E and Supplementary Fig. S6B).

Finally, to further investigate the importance of HIF1α in the radiation-induced increase of JMY, we assessed JMY mRNA expression in HIF1α knockdown TG1N and TG16 GSCs (Supplementary Fig. S5C and S6C) and their respective controls 18 h after irradiation (0.5 Gy). As shown in Fig. 4F and Supplementary Fig. S6D, JMY mRNA expression increased after irradiation in shCt GSCs cells, but remained unchanged in both HIF1α knockdown TG1N and TG16 GSCs. Therefore these data clearly demonstrate that the transcriptional activation of JMY is dependent on HIF1α in irradiated GSC.

We next investigated the effects of JMY knockdown using a specific siRNAs that decreased by 64 and 70% JMY mRNA expression in TG1N and TG16 cells respectively compared to siCt-transfected cells (Supplementary Fig. S6E). JMY knockdown did not alter the basal migration rates of TG1N (Fig. 4G) and TG16 cells (Supplementary Fig. S6F). In contrast, it abolished the effects of radiation on their migration (Fig. 4G and Supplementary Fig. S6F).

We next obtained TG1N cells stably knocked down for JMY using lentiviral vectors expressing both GFP and shRNAs against JMY. JMY mRNA expression was decreased by 72% in shJMY-TG1N, as compared to cells expressing a negative control shRNA (shCt-TG1N cells) (Fig. Supplementary S6G). As reported above using siJMY, the JMY knockdown did not alter the in vitro basal migration rates of TG1N, whereas it completely abolished the radiation-induced migration (Fig. 4H). The stable JMY knockdown prevented also the radiation-induced invasion capacity of TG1N cells, as estimated in the invasion chamber test (Fig. 4I).

ShJMY-TG1N cells and shCt-TG1N cells were then stereotaxically injected into the striatum of nude mice just after irradiation as described above. Analysis of serial coronal brain slices obtained two days after engraftments revealed that contrary to shCt-TG1N cells, 0.5 Gy radiation prior to injection did not increase the dispersion of shJMY-TG1N cells (Fig. 4J).

Altogether, these results demonstrate that radiation-induced migration of GSCs is linked to HIF1α-dependent cytoplasmic accumulation of JMY.

**Figure 5:**
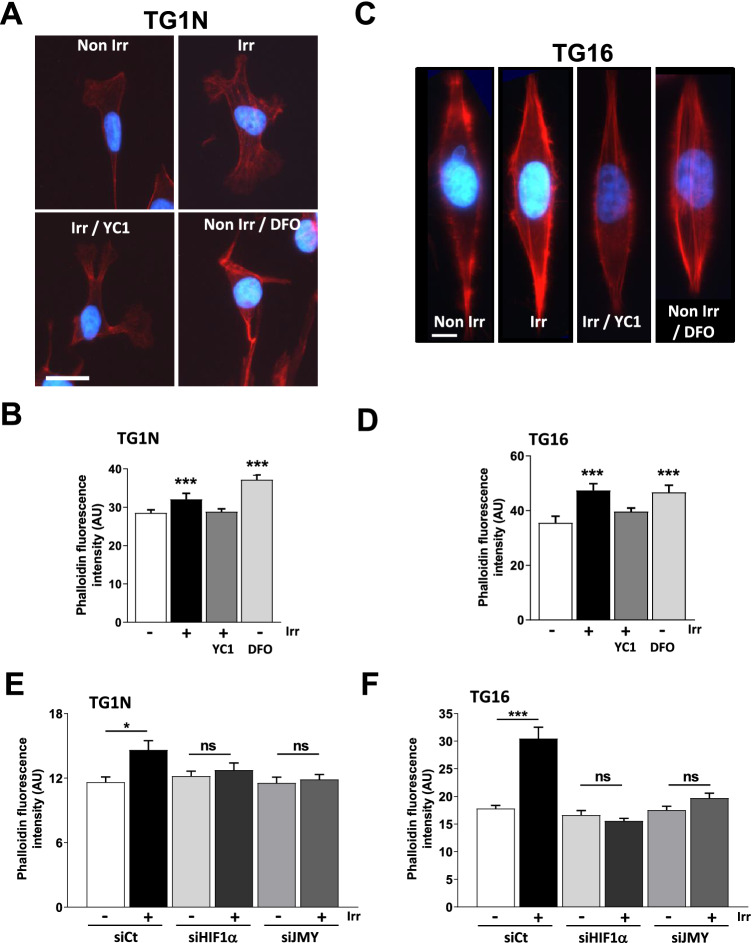
Irradiation increases cellular levels of F-actin in a JMY-dependent manner. (**A**,**C**) F-actin staining with phalloidin in TG1N (**A**) or in TG16 (**C**) GSCs. Scale bars: 20 µM (**A**) and 10 µM (**C**). (**B**,**D**) Quantification of phalloidin fluorescence intensity 24 h after 0.5 Gy irradiation (in cells pretreated or not with 50 µM YC1) or after 100 µM DFO for TG1N (**B**) and TG16 GSCs (**D**). At least 35 cells were scored per condition (***p < 0.001). (**E**,**F**) Quantification of F-actin fluorescence intensity staining by phalloidin after irradiation (0.5 Gy) or not in GSCs with siCt, siJMY or siHIF1α-electroporated TG1N (**E**) and TG16 (**F**). At least 20 and 25 cells were scored by condition (respectively in TG1N and TG16 GSCs; *p < 0.05, ***p < 0.001 and *ns* not significant).

The role of JMY in cell motility has been attributed to its actin nucleation-promoting activity^46,48^. We thus quantified F-actin in irradiated GSCs by measuring Alexa-596 phalloidin staining. Interestingly, 24 h after irradiation (i.e*.* at the peak of radiation-induced migration (Fig. 1), we showed a significant increase in cellular content of F-actin in irradiated, as well as DFO-treated GSCs (Fig. 5A–D). By contrast, HIF1α inhibition by YC1 (Fig. 5A–D) or by siRNAs (Fig. 5E,F), as well as the knockdown of JMY (Fig. 5E,F), prevented both the increase of F-actin and the radiation-induced migration (Figs. 3E and 4G, Supplementary Fig. S4G and S6F).

Altogether, our data demonstrate that ionizing radiation at sublethal dose enhances the migration of human GSC via the HIF1/JMY pathway involving the nucleation promoting activity of JMY.

### Radiation-induced migration is related to GSC stemness

**Figure 6:**
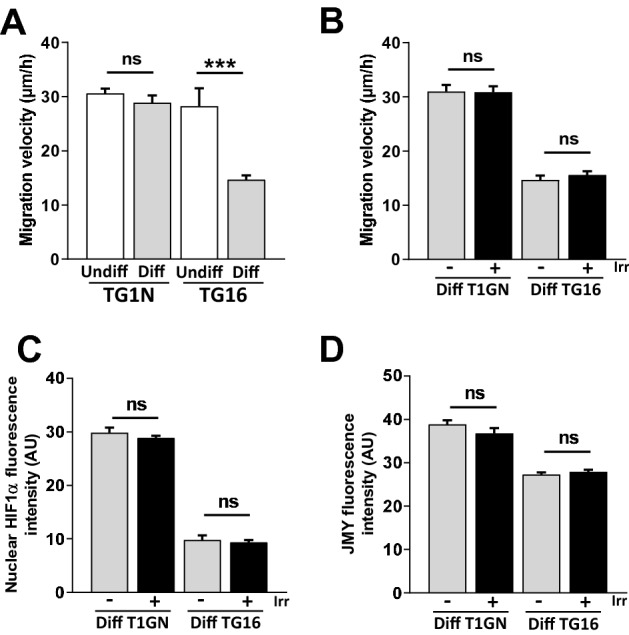
Radiation-induced migration is related to stemness. (**A**) Differentiation markedly reduced the motility of TG16 cells. The mean migration velocities of GSCs (Undiff) and differentiated cancer cells (Diff) were calculated from at least 75 cells per group 24 h after plating (***p < 0.001 and *ns* not significant). (**B**) Irradiation did not enhance the migration of differentiated cancer cells. Cells were tracked 24 h after irradiation (0.5 Gy) every ten minutes over the course of 4 h. The mean migration velocities of control (0 Gy) and irradiated cells were calculated from at least 120 cells per group tracked every ten minutes over the course of 4 h (*ns* not significant). (**C**) Irradiation did not increase nuclear expression of HIF1α in differentiated cells. Immunostainings were performed 1 h after irradiation (0.5 Gy). Nuclear HIF1α fluorescence intensity was measured in at least 50 cells per condition (ns: not significant). (**D**) Irradiation did not increase JMY expression in differentiated cells. Immunostainings were performed 24 h after irradiation (0.5 Gy). JMY fluorescence intensity was measured in at least 65 cells per condition (*ns* not significant).

We finally investigated the dynamic behavior of our cell lines cultured under differentiating (diff) conditions (medium supplemented with 10% FBS without FGF2 and EGF) that let them loss their stem cell properties including their capacity to generate brain tumors in immunodeficient mice^29^. No obvious morphological changes were observed in diffTG1N which maintained a stable (diffTG1N) migration velocity compared to their parental cells (Fig. 6A). In contrast, diffTG16 cells presented with a markedly flattened cytoplasm and a significant decrease in migration velocity compared to the parental GSC lines (Fig. 6A).

Interestingly, ionizing radiation did not increase migration velocity (Fig. 6B) nor the expression of HIF1α (Fig. 6C) and JMY (Fig. 6D) in the differentiated cell lines, suggesting that the radiation-induced stimulation of cell motility is specific to GSCs due to the lack of activation of the HIF1α/JMY pathway in differentiated cells.

## Discussion

GSCs are thought to play crucial roles in GBM relapse^23,25^. Here, we report that sublethal doses of irradiation enhance the motility and invasiveness of human GSCs through HIF1α nuclear accumulation that in turn increases the cytoplasmic actin nucleator JMY. Our study is thus in line with previous reports showing that ionizing radiation promotes migration and invasion in various cancer cell lines^15^, including human glioma cell lines^5–14,16,17,49^. However, to our knowledge, our study is the first to show not only that human GSCs are prone to this radiation effect, but also that this is specifically linked to their stemness properties, as radiation did not induce this effect on “differentiated” glioma cells. This link may explain why we were able to use a lower dose (0.5 Gy) to enhance GSC motility compared to the dose of 1 Gy^10–12^ or greater^5–9,13,14,16,17^ required to stimulate non-stem-like human glioma cell lines.

Previous studies have reported that hypoxia may increase the migration/invasion capacity of various glioblastoma cell lines in a HIF1α-dependent manner^31–35,50^, suggesting that HIF1α is involved in therapeutic failure and GBM relapse^49^. Our study shows that the key factor linking the radiation-induced enhancement of GSCs to stem-like properties is the fact that GSCs are much more prone than differentiated cells to the activation of HIF1α in response to radiation. This may also explain why we were able to show that 0.5 Gy enhanced the migration of GSCs, whereas Kim et al. have reported that 6 Gy-irradiation in either single or fractioned doses, but not 2 Gy irradiation, was able to induce HIF1α stabilization and stimulate the migration of U87 and U373 glioma cells^9^.

We have shown that radiation induces the migration of GSCs through a HIF1α-dependent cytoplasmic accumulation of JMY. Indeed, whereas we cannot exclude the involvement of other HIF1α-dependent pathway due to the well-known pleiotropic effects of HIF1α, our data demonstrate that JMY was absolutely required for this radiation-induced effect. JMY was initially described as a transcriptional cofactor cooperating with p300/CBP to augment p53 signaling during the DNA damage response^27,51^. JMY has been reported to accumulate in the nucleus after exposure to ultraviolet light, etoposide and actinomycin, promoting p53-mediated apoptosis. Since TG1N, but not TG16^52^, are p53 proficient, the role of JMY in radiation-induced migration is not related to p53. Moreover, the ionizing radiation doses used in this study did not trigger the nuclear accumulation of JMY and induced very low levels of GSC apoptosis, suggesting that JMY does not act as a transcriptional cofactor in radiation-induced cell migration. Rather, our data show that JMY is functioning through its previously described role in cell motility under hypoxic conditions by controlling actin dynamics via its nucleation-promoting activity^46,47^.

HIF1α has been widely considered a prominent cancer drug target due to its role in the regulation of multiple survival pathways in solid hypoxic tumors. However, targeting HIF1α is highly challenging and may induce severe side effects due its multiple functions^53–56^. In this context, specific targeting of JMY could provide new therapeutic perspectives to limit radiation-induced migration of GSCs and hence prevent tumor recurrence following radiotherapy.

## Materials and methods

### Human glioma stem-like cell (GSC) lines and treatments

The TG1N and TG16 GSC lines were obtained from surgical resections carried out at Sainte Anne Hospital (Paris, France) on patients with high-grade gliomas according to the WHO classification^28,29,52^. Since then they were systematically cultured as tumorospheres in defined stem cell culture condition (serum-free Dulbecco’s Modified Eagle Medium DMEM/F12 supplemented with B27 without vitamin A (1X, Invitrogen), heparin (5 µg/mL, Stem Cell Technologies), human recombinant epidermal growth factor (EGF, 20 ng/ml, Sigma) and human basic fibroblast growth factor (FGF-2, 20 ng/ml, Sigma)) at 37 °C in an atmosphere containing 5% CO_2_. Every week, cells were mechanically dissociated after a 10 min incubation at room temperature with the Accutase cell dissociation reagent (Sigma) and reseeded at 0.5 × 10^6^ cells per T75 flask.

Cells were γ-irradiated with the indicated doses 24 h after plating using a ^137^Cs irradiator (IBL637, CIS BIO International or GSR-D1, Gamma-Service Medical GmbH) or with a ^60^Co medical irradiator (Alcyon). When indicated, Deferoxamine (DFO, Interchim), or YC1 (Cayman Chemical) was added 2 h before irradiation.

### Time-lapse experiments

Cells were plated on 24-well plates (25–35,000 cells /well) coated with laminin (5 µg/mL; Sigma). Videomicroscopy was carried out at least 8 h after plating using an inverted microscope (Olympus IX81) coupled with a Coolsnap HQ camera (Princeton Instruments) controlled with Metamorph software (Universal Imaging) as previously described^57^ or using a NIKON A1R confocal laser microscope (Nikon Corp. Tokyo). Imaging conditions were maintained at 37 °C (The Box & The Cube, LIS), 5% CO_2_ and 18% O_2_ with a relative humidity of 95% controlled by an active gas supply system (the Brick, LIS). Images were taken of 6–20 fields per condition using a 10X objective (Olympus IX81) or in mosaic acquisition with a 20X objective (Nikon A1R) every 10 min. Tracking and overlay of individual cell tracks over a period of 4 h were carried out using the track object function in Metamorph software (Molecular Devices) or using the MTrackJ plugin in ImageJ software. Dynamic parameters as migration velocity, mean square displacement (MSD) and directional persistence were calculated with an Excel macro developed by F. Cordelières (Bordeaux imaging center, UMS 3420 CNRS, France) and/or with the custom-made open-source computer program DiPer^58^.

### Cell cycle analysis

GSCs were collected after accutase treatment, washed with PBS, fixed in 70% ice-cold ethanol and kept at − 20 °C for 24 h. Fixed GSCs were then washed in PBS and resuspended in propidium iodide and RNase (50 µg/ml each). The cell suspension was incubated for 15 min at 37 °C and cell cycle data was obtained by flow cytometry (LSRII; BD Biosciences) with CellQuest software. Cell cycle distribution was analyzed by using the univariate cell cycle platform in Flow Jo V10 software, the Den Jett Fox model integrated (Tree Star, USA).

### Cell viability

Cell viability was estimated by videomicroscopy experiments as described in time-lapse experiments section (see above). Just before the beginning of acquisition, the IncuCyte Cytotox Reagent was added in the full media at the concentration of 250 nM as recommended by the manufacturer (Essen Bioscience). Once cells become unhealthy, the plasma membrane integrity diminishes, allowing entry of the IncuCyte Reagent and yielding a 100–1000-fold increase in fluorescence upon binding to DNA. Cytotoxity was estimated by the ratio of dead cells number (red fluorescent cells) on viable cells over time at 8, 12, 16, 20, 24 and 28 h after irradiation at the doses between 0 to 3 Gy in a mosaic of 2 X 6 fields (objective 20X). Data were obtain in triplicate from two independent experiments. Percentage of cell death was estimated by the number of red-positive cells dead on total cell number counted automatically with NIS software.

### Cell invasion assay

Experiments were performed using Matrigel invasion chambers (BD Biosciences) as described previously^29^. Briefly, 24 h after irradiation (0.5 Gy), cell suspensions (5 × 10^4^ cells/0.5 mL) were seeded in triplicate onto the upper chamber with medium lacking growth factors (EGF and FGF). Culture medium with growth factors was added to the lower chamber. After 29 h at 37 °C, cells still present on the upper surface of the membrane were gently removed with cotton-tipped swabs. Cells that have invaded the lower surface of the membrane were fixed with 4% paraformaldehyde and their nuclei were stained with DAPI (1 µg/ml; Sigma). Membranes were imaged with a Nikon eclipse 50i (objective 10X/NA 0.3) and a Hamamatsu CCD ORCA-05G camera controlled by NIS-element BR V3.2 software equipped with a motorized stage. Nuclei or GFP-positive cells—in experiments involving cells transfected with lentiviral vectors—detected on membranes were numbered and results were expressed as percentage of unirradiated controls.

### HIF1α and JMY knockdowns

After dissociation, cells were electroporated (1300 V, 10 ms, 3 pulses) with pools of 4-target-specific siRNAs targeting HIF1α (ON-TARGETplus SMART pool human HIF1α, L-004018-00, Dharmacon) or of 3 target-specific siRNAs to JMY (siJMY, SC35724, Santa Cruz) using the Neon Transfection System (Life Technologies) according to the manufacturer’s instructions. Negative control (siCt, Santa Cruz SC37007 or ON-TARGETplus non targeting pool; D-001810-10-20) were simultaneously used to evaluate RNAi off-target effects and verify the accuracy of gene specific siRNA dependent RNAi. Transfected cells were then plated on laminin-coated 24-well plates. Knockdown efficiency was assessed 24–48 h after transfection by RT-qPCR.

Generation of stable Hif1α-deficient GSCs was performed using a pTRIP-MND-GFP-H1-SanDI lentivirus vector^59^ in which a shRNA targeting 5′- GTGATGAAAGAATTACCGAAT -3′ of HIF1α (NM_181054.1, NM_001530.2; hypoxia-inducible factor 1α subunit) was inserted. The target sequence was available in a database of National RNAi Core Facility of BROAD Institute and the plasmid lentivirus shHIF1α was kindly provided to us by Pflumio’s laboratory (INSERM U1274, CEA, DRF-JACOB-IRCM-SCSR-LSHL, UMR Genetic Stability Stem Cells and Radiation).

Generation of stable JMY-deficient GSCs was performed using lentivirus vectors constructed as follow: different hybrids of primers encoding the expected shRNA sequences were annealed by mixing 500pmoles each in NEB2.1 1 × buffer (New England Biolabs, Ipswich, MA). After 5 min at 100 °C, the annealing occurred slowly by cooling down the heat block for overnight in a styrene box. Thus, the different hybrids of primers were inserted in pTRIP-MND-GFP-H1-SanDI^59^ under control of H1 promoter by complementary single-strand annealing^60^. Briefly, the plasmid was digested by *San*DI (ThermoFisher Scientific, Waltham, MA) and was treated with T4 DNA polymerase (0.75U) for generating single-strand sequence complementary to single strand extended sequences present in primer hybrids. The annealing reaction was transformed in DH5α-T1R homemade competent cells. Positive clones were validated by DNA sequencing.

A pTRIP-H1-MND-GFP lentiviral vector expressing a small hairpin directed against HVC (5′- GTGTTGGGTCGCGAAAGG -3′)^61^ was used as control (shCt).

One day after platting on laminin substrate (5 µg/mL; Sigma), GSCs were transduced with a pool of the three lentiviral vectors at a MOI 5 (MOI-defined as the number of lentiviral particles able to transduce used per HEK-293). Transduced GSCs expressing GFP were then Fac-sorted based on GFP expression and thereafter maintained in culture.

### Reverse transcription–quantitative PCR (RT-qPCR)

RNA was extracted using the RNeasy Plus Mini Kit or the RNeasy plus Micro Kit (Qiagen) according to the manufacturer’s instructions. Isolated RNAs were transcribed into cDNA using the High capacity RNA to cDNA Master Mix (Applied Biosystems). Quantitative PCR reactions were performed in 96-well plates in triplicate using SYBR Green Master Mix (Applied Biosystems). The primers used are listed in Supplemental Table S4.

### Luciferase JMY reporter assay

GSCc (15 × 10^3^ cells) were electroporated using the Neon® transfection system (Thermo Fisher Scientific) with either 50 ng of the control (empty) pLightSwitch_empty_Prom vector (ref #S790005) or the pLightSwitch Prom reporter plasmid for the JMY gene promoter (#S719700; SwitchGear Genomics), then immediately transferred in 96-well plates previously coated with laminin (5 µg/mL; Sigma). GSCc were irradiated (0.5 Gy) 24 h after electroporation and Luciferase reporter activity was determined at different time points using the LightSwitch Dual Assay System (SwitchGear Genomics) according to the manufacturer's instructions.

### Sub-cellular fractionation and western blot

Cytoplasmic and nuclear protein extraction and protein quantification was performed according to the manufacturer’s recommendations (CelLytic™ NuCLEAR™ Extraction Kit, MERCK and Pierce™ BCA Protein Assay Kit, ThermoFisher respectively). Proteins were separated by PAGE and transferred following standard protocols^62^. Membranes were first probed using the following primary antibodies: anti-HIF1α (BD610958, clone 54 from Bioscience; 1/500), anti-Lamin B1 (sc374015, Santa Cruz; 1/500) or anti-α-tubulin (DM1A, Sigma; 1/4000). Next, membranes were incubated with secondary IRDye680 and IRDye800 antibodies (respectively 926-68021 and 926-32211, Licor; 1/15000). Bands were detected with the Odyssey Infrared Imaging System (Licor) and quantified using ImageStudio Lite 5.2 software (Licor).

### Intracerebral grafts

Swiss^nu/nu^ mice were maintained with access to food and water ad libitum in a colony room kept at a constant temperature (19–22 °C) and humidity (40–50%) on a 12:12 h light/dark cycle. All animal-related procedures were performed in compliance with the European Communities Council Directive of 22th September 2010 (EC/2010/63) and were approved by Comité d’Ethique en Expérimentation Animale, Direction de la Recherche Fondamentale, CEA (authorization #A12-029 and #A16-002; CEtEA-CEA DRF IdF).

Cell grafting was performed as previously described^63^ using a stereotaxic apparatus (David Kopf model 900 Small Animal Stereotaxic Instrument). 100,000 dissociated cells (2 µL) were inoculated into the two hemispheres using a 33G Hamilton needle (Hamilton Bonaduz) at the following coordinates: anteroposterior: + 0.5 mm, dorsoventral: − 3 mm and lateral: + 1.5 (control cells) and − 1.5 mm (irradiated cells). Forty-eight hours after grafting, animals were deeply anesthetized and intracardially perfused with 4% paraformaldehyde in PBS. Brains were removed, postfixed overnight, cryoprotected with 10% sucrose/PBS, and frozen in dry ice-cooled isopentane. A cryostat (Leica CM3050S) was used to prepare serial coronal brain sections (14 µm) with an inter-slice spacing of 60 µm. These sections were mounted in order to analyze the dispersion of grafted cells by immunofluorescence staining with an anti-human nestin antibody (MAB1259, 1/400; R&D Systems, Fig. 2b) or immunodetection of GFP expression as previously described^29,63^. Images were acquired at 10 × magnification using NIS Elements software with a Pathfinder-Nikon motorized microscope (Nikon Instruments Inc.).

GSC dispersion in the coronal plane was calculated as the sum of the surfaces in µm^2^ occupied by human nestin-positive or GFP-positive cells in the different coronal slices analyzed.

### Immunostaining

Adherent cells were fixed for 10 min in 4% paraformaldehyde in PBS and then permeabilized in 0.1% Triton X-100 in PBS as previously described^29^. Cells were then incubated with the primary antibody in blocking buffer for 1 h at room temperature and then washed and incubated with an Alexa-conjugated secondary antibody (1/1000, Molecular Probes) and with 2 Units of AlexaFluor 594 phalloidin (Thermo Fisher scientific) per coverslip for one hour. Cells were counterstained with DAPI (1 µg/ml, Sigma) and mounted with Fluoromount (Southern Biotech). The primary antibodies used were rabbit anti-HIF1α (NB100-449, Novus Biological), mouse anti-HIF1α (NB100-105, Novus Biological) and MA1-516, Thermo Fisher Scientific), goat anti-JMY (L16, Santa Cruz), mouse anti-JMY (G11, Santa Cruz) and rabbit anti-JMY (M300, Santa Cruz). Images were captured using a BX51 (Olympus) coupled with a Retiga200R camera or using a Leica TCS SPE confocal microscope (Leica Microsystems). Nuclear HIF1α, cytoplasmic JMY and F-actin mean fluorescence intensities were measured using DAPI or phalloidin staining for object segmentation with ImageJ software.

### Statistical analyses

All values are reported as the mean ± SEM. Statistical significance for two groups was assessed by the unpaired Mann–Whitney test or t-test. For comparison between more groups, a non-parametric ANOVA was performed followed by post hoc tests. As previously reported^64^, a two-way ANOVA with time and condition was used to compare MSD data. Statview (Abacus Concepts) and Prism Graphpad 7.1 software programs were used. Statistical significance levels are denoted as follow: *p < 0.05, **p < 0.01 and ***p < 0.001.

## Supplementary information

Supplementary Information 1.

Supplementary Figures.

Supplementary Video 1.

Supplementary Video 2.
