# Supplementary material for: The HIF1α/JMY pathway promotes glioblastoma stem-like cell invasiveness after irradiation
Source: Sci Rep. 2020 Oct 30;10:18742. doi: 10.1038/s41598-020-75300-5 (PMC7603339; doi:10.1038/s41598-020-75300-5)
Supplement: Supplementary file 1 — Supplementary Information 1. [file 41598_2020_75300_MOESM1_ESM.docx]

**The HIF1α/JMY pathway promotes glioblastoma stem-like cell invasiveness after irradiation**

Laurent R. Gauthier ^1,*^, Mahasen Saati ^1^, Hayet Bensalah-Pigeon ^1^, Karim Ben M’Barek ^1,2^, Oscar Gitton-Quent ^1^, Romane Bertrand ^3^, Didier Busso ^1^, Marc-André Mouthon ^1^ , Ada Collura ^3^, Marie-Pierre Junier ^4^, Hervé Chneiweiss ^4^, José R. Pineda ^1,5^, and François D. Boussin ^1,*^

^1^ Université de Paris and Université Paris-Saclay, Inserm, LRP/iRCM/IBFJ CEA, UMR Stabilité Génétique Cellules Souches et Radiations, F-92265, Fontenay-aux-Roses, France.

^2^ Present address: INSERM U861, I-Stem, CECS, UEVE, AFM, Institute for Stem Cell Therapy and Exploration of Monogenic Diseases, 91100 Corbeil-Essonnes, France

^3^ Sorbonne Université, UPMC Univ Paris 06, Inserm, UMRS 938, Équipe Instabilité des microsatellites et cancer, Centre de recherche Saint Antoine, 75012 Paris, France.

^4^ CNRS UMR8246, Inserm U1130, Neuroscience Paris Seine-IBPS, UPMC, Sorbonne Universités, Paris, France

^5^ Present address: Achucarro Basque Center for Neuroscience, Sede Building, E-48940 Leioa, Vizcaya, Spain

*** Corresponding authors:**

François D. Boussin

LRP-UMR E008/U1274 CEA 18 route du panorama, 92265 Fontenay-aux-Roses, France

Phone: (+33) 1 46 54 97 91 / 91 45

email: boussin@cea.fr

Laurent R. Gauthier

LRP- UMR E008/U1274 CEA, 18 route du panorama, 92265 Fontenay-aux-Roses, France

Phone: (+33) 1 46 54 91 45

email: laurent-r.gauthier@cea.fr

**SUPPLEMENTARY TABLES**

**Supplementary Table S1: Apoptosis 24 hours post-irradiation in GSCs is not significant until the irradiation dose of 1.5 Gy.** Fold induction of Caspase 3/7 in irradiated GSCs 24 hours post-irradiation (0.5 Gy) was measured using the Apo-ONE® Homogeneous Caspase-3/7 Assay according to the manufacturer’s instructions (**p < 0.01).

|  | 0 Gy | 0.5 Gy | 1.5 Gy | 3 Gy |
| --- | --- | --- | --- | --- |
| TG1N | 1.00 ± 0.01 | 1.09 ± 0.01 | 1.07 ± 0.04 | 1.15 ± 0.01 ** |
| TG16 | 1.00 ± 0.01 | 1.10 ± 0.02 | 1.10 ± 0.03 | 1.13 ± 0.01 |

**Supplementary Tables S2:** Percentage of dead TG1N and TG16 cells monitored between 8 and 28 hours post-irradiation by videomicroscopy using IncuCyte Cytox Reagent according to manufacturer’s instructions. Percentage of dead cells detected by red-fluorescence was estimated at 8, 12, 16, 20, 24 and 28 hours after irradiation at the doses from 0 to 3 Gy in a mosaic of 2 X 6 fields (objective 20X) from at least 400 cells from two videomicroscopy experiments performed in triplicates. Comparisons were performed between respective unirradiated controls and irradiated cells for the indicated time point after irradiation (*p < 0.05 and **p < 0.01).

| TG1N | 0 Gy | 0.1 Gy | 0.25 Gy | 0.5 Gy | 1.5 Gy | 3 Gy |
| --- | --- | --- | --- | --- | --- | --- |
| 8 hrs | 0.56 ± 0.09 | 0.94 ± 0.21 | 1.02 ± 0.30 | 0.72 ± 0.35 | 1.31 ± 0.23 | 1.12 ± 0.18 |
| 12 hrs | 0.85 ± 0.15 | 1.27 ± 0.24 | 1.39 ± 0.18 | 0.82 ± 0.14 | 1.11 ± 0.18 | 1.00 ± 0.08 |
| 16 hrs | 1.15 ± 0.27 | 1.23 ± 0.29 | 1.17 ± 0.15 | 1.06 ± 0.18 | 1.24 ± 0.14 | 0.98 ± 0.12 |
| 20 hrs | 0.99 ± 0.24 | 1.25 ± 0.32 | 1.49 ± 0.26 | 1.07 ± 0.18 | 1.45 ± 0.14 | 1.10 ± 0.15 |
| 24 hrs | 1.01 ± 0.16 | 1.12 ± 0.27 | 1.09 ± 0.16 | 1.10 ± 0.14 | 1.25 ± 0.11 | 1.07 ± 0.08 |
| 28 hrs | 1.09 ± 0.15 | 1.04 ± 0.23 | 1.36 ± 0.09 | 1.32 ± 0.19 | 1.24 ± 0.09 | 1.03 ± 0.10 |

| TG16 | 0 Gy | 0.1 Gy | 0.25 Gy | 0.5 Gy | 1.5 Gy | 3 Gy |
| --- | --- | --- | --- | --- | --- | --- |
| 8 hrs | 0.79 ± 0.23 | 0.77 ± 0.20 | 0.58 ± 0.21 | 1.07 ± 0.20 | 0.47 ± 0.12 | 0.86 ± 0.40 |
| 12 hrs | 0.84 ± 0.20 | 0.89 ± 0.14 | 0.65 ± 0.13 | 1.02 ± 0.12 | 0.60 ± 0.09 | 0.68 ± 0.14 |
| 16 hrs | 1.35 ± 0.26 | 1.68 ± 0.17 | 1.09 ± 0.21 | 2.43 ± 0.15 | 1.27 ± 0.34 | 1.48 ± 0.20 |
| 20 hrs | 2.17 ± 0.37 | 2.14 ± 0.23 | 1.49 ± 0.32 | 4.67 ± 0.38* | 2.47 ± 0.47 | 2.40 ± 0.36 |
| 24 hrs | 2.13 ± 0.35 | 3.04 ± 0.24 | 2.41 ± 0.57 | 5.44 ± 0.55** | 3.40 ± 0.58 | 2.97 ± 0.36 |
| 28 hrs | 2.87 ± 0.41 | 3.90 ± 0.30 | 2.83 ± 0.58 | 5.86 ± 0.56* | 3.65 ± 0.65 | 3.81 ± 0.55 |

**Supplementary Tables S3**: **Cell cycle analysis of TG1N and TG16 GSCs 24 hours after γ-irradiation**. Percentages of viable cells in the different phases of the cell cycle (*p < 0.05).

| TG1N | | | |
| --- | --- | --- | --- |
|  | G1 | S | G2/M |
| 0 Gy (n=6) | 68.1 ± 0.7 | 7.3 ± 0.3 | 24.6 ± 0.5 |
| 0.5 Gy (n=5) | 66.9 ± 1.7 | 6.5 ± 0.9 | 26.5 ± 1.3 |
| 3 Gy (n=3) | 50.3 ± 1.0 * | 5.4 ± 0.3 * | 44.3 ± 0.8 * |

| TG16 | | | |
| --- | --- | --- | --- |
|  | G1 | S | G2/M |
| 0 Gy (n=6) | 58.0 ± 0.3 | 12.7 ± 0.8 | 26.9 ± 2.5 |
| 0.5 Gy (n=5) | 60.5 ± 4.3 | 9.7 ± 1.2 | 30.1 ± 2.7 |
| 3 Gy (n=3) | 52.3 ± 0.2 * | 9.6 ± 0.7 | 38.0 ± 0.5 * |

**Supplementary Table S4: Primers used for quantitative Real-Time PCR**

| **Primers** | **Couple** | **Sequences** |  |
| --- | --- | --- | --- |
| **HIF1α** | F1-60  R1-61 | Sens: TGCAACATGGAAGGTATTGCAC  Antisens: GCACCAAGCAGGTCATAGGT |  |
| **JMY** | F3-68  R3-69 | Sens: GCCAAGAGAGACAGAGAAC  Antisens: GCATGAGCTAGTCGTAATC |  |
| **GUS** | F2-96  R2-97 | Sens: ACCTCCAAGTATCCCAAGG  Antisens:ACAGAAGTACAGACCGCTG |  |
| **BNIP3** | F1-251  R1-252 | Sens: CCTTCCATCTCTGCTGCTCTC  Antisens : CGCCTTCCAATATAGATCCCCAAT |  |
| **GLUT1** | F1-247  R1-248 | Sens: TGGCATCAACGCTGTCTTCT  Antisens : AGCCAATGGTGGCATACACA |  |
| **VEGFA** | F2-92  R2-93 | Sens: AAAAACACAGACTCGCGTTGC  Antisens : GCTTGTCACATCTGCAAGTACG |  |

**Supplementary Movies S1 and S2**: **Dynamics of TG1N (Movies S1) and TG16 (Movies S2) over time.**

Each video was recorded using a NIKON A1R confocal laser microscope twenty-four hours after plating cells on a laminin substrate. Images were acquired in mosaic acquisition mode with a 20X objective every 10 min over the course of 240 minutes. Video editing was performed with ImageJ software and saved in AVI format at a frame rate of 10 fps.

**SUPPLEMENTARY FIGURE LEGENDS**

**Supplementary Figure S1.**

**TG1N and TG16 have the capacity to generate intracerebral tumors in nude mice and have a high velocity cultured on laminin substrate**

(A) Representative engraftments of luciferase-expressing GSCs (10^5^ cells) into the striatum of Swiss Nu/Nu mice 24 weeks after engraftment (TG1N or TG16). GSCs were transduced using a lentiviral vector containing a Luciferase cassette (pTRIP-MND-Luciferase-Ires-GFP) and grafted (10^5^ cells) into the striatum of female Swiss Nu/Nu mice. Tumor growth was followed after D-Luciferin administration using an IVIS Lumina III In Vivo Imaging System (PerkinElmer). Tumor formation was observed in all GSCs grafted mice (n ≥3 for each GSC line).

(B) Typical microscopic phase contrast pictures of TG1N and TG16 GSC lines 24 hours after plating on a laminin substrate. Contrary to TG1N GSCs that mainly adopted a bipolar and elongated shape (Supplementary video 1), TG16 GSCs were morphologically heterogeneous forming two distinct populations with a majority of bipolar and elongated flattened shape cells and a minority of spherical and blebbing morphology cells (34.3 ± 1.6%, white arrow). Importantly, TG16 GSCs could alternate between these two distinct morphologies (Supplementary Video 2). Scale bar: 50 µM

(C) Mean migration velocity of bipolar/elongated cells and blebbing GSCs derived from GBM (TG1Nand TG16) plated on laminin substrate. The mean velocity of TG16 bipolar cells was similar to that of TG1N cells whereas TG16 blebbing cells migrated at a significantly slower velocity than their respective bipolar counterparts (14.15 ± 0.9 µm/h, ^***^p<0.001). TG16 bipolar cells migrated with no predefined direction, in a manner similar to TG1N cells (Supplementary Movies 1 and 2). Further analyses were thus only performed on the bipolar elongated sub-population. Human GSCs adopt a random motility pattern with a high velocity on laminin substrates. Mean migration velocities were calculated from monitoring of 224 TG1N cells, 45 bipolar TG16 cells, and 68 blebbing TG16 cells every ten minutes over the course of 4 hours (***p < 0.001 and ns: not significant). Similar results were obtained from three independent experiments.

**Supplementary Figure S2.**

**Effects of ionizing radiation on TG16 clonogenicity.**

(A) Representative images of clonogenicity assay. Briefly, TG16 cells were plated in 6-well plates at 300 cells/well and irradiated (0 to 3 Gy) 24 hours later then switched to fresh media and allowed to grow for 13 days. They were then fixed in acetic acid/methanol (1:7) and stained with crystal violet.

(B and D) Colonies were counted to determine the plating efficiency for each dose (PE = number of colonies formed/number of seeded cells x 100). Plating efficiencies were expressed as mean ± SEM from three experiments (*p < 0.05) for TG16 (B) and TG1N (D)**.**

(C and E) The surviving fraction for each dose is the ratio of PE of irradiated sample / PE of unirradiated control (Munshi et al., 2005). Data were expressed as mean ± SEM obtained in three experiments (*p < 0.05) for TG16 (C) and TG1N (E).

**Supplementary Figure S3.**

**Irradiated GSCs explore a wider territory without alteration of their directional persistence.**

(A and B) Irradiated (0.5 Gy) TG1N (red, A) and TG16 GSCs (red, B) significantly explored a larger territory than their respective unirradiated controls (blue). The figures show the mean square displacement (MSD, µm^2^), a measure of area explored by cells for 10 min intervals, calculated from 24 to 26 hours post-irradiation. Two-ANOVA with time and conditions: p < 0.001 for the two cell lines.

(C, D, E and F) The directional persistence of TG1N (C and E) and TG16 (D and F) are not affected by irradiation (0.5 Gy, C and D). Directional persistence calculated from 24 to 28 hours either by the end-point method (C and D) or over time (every 10 minutes, E and F).

MSDs, directional persistence and directional ratio over time were generated using the custom-made open-source computer program DiPer (Gorelik and Gatreau, 2014), from tracking of 223 unirradiated and 214 irradiated TG1N cells and 172 unirradiated and 175 irradiated TG16 cells (ns: not significant).

**Supplementary Figure S4.**

**Nuclear accumulation of HIF1α is involved in radiation-induced migration of GSCs.**

(A, B) Quantification of nuclear HIF1α fluorescence intensity in irradiated (0.5 Gy) TG1N cells (A) and irradiated (0.5 Gy) TG16 cells (B) over time (n = 50 cells per time-point; *p < 0.05 and **p < 0.01).

(C) RT-qPCR quantification of HIF1α mRNA in TG1N cells at different times after irradiation (0.5 Gy). Each time-point was performed in duplicates.

(D) YC1 prevents the radiation-induced nuclear accumulation of HIF1α in TG16 cells. TG16 cells were either treated with 50 µM YC1 and irradiated 2 hour later or treated with 100 µM DFO. After 1 hour, nuclear HIF1α fluorescence intensity was measured in at least 50 cells per condition (**p < 0.01 and ***p < 0.001).

(E) YC1 prevents the radiation-induced cytoplasmic accumulation of JMY, while DFO stimulates the cytoplasmic accumulation of JMY. TG16 cells were treated with 50 µM YC1 one hour before irradiation (0.5 Gy). Migration velocity was measured 24 hours after irradiation or after treatment with 100 µM DFO (n = 120 cells per condition; **p < 0.01 and ***p < 0.001).

(F) mRNA levels of HIF1α in TG1N cells (left panel) and TG16 cells (right panel) transfected with a siRNA targeting HIF1α (siHIF1α) or a scrambled control siRNA (siCt). Each condition was performed in triplicates (***p < 0.001).

(G) Migration velocity of TG16 cells transfected with siHIF1α or siCt 24 hours after 0 or 0.5 Gy irradiation (n = 100 cells per condition; **p < 0.01).

**Supplementary Figure S5.**

**YC1 inhibits expression of HIF1α target genes in irradiated-GSCs and HIF1α nuclear accumulation induced by DFO.**

(A) RT-qPCR quantification of mRNA coding for well-known HIF1a target genes BNIP3, GLUT1 and VEGFA in TG1N cells previously treated or not with 50 µM YC1 two hours before irradiation (0.5 Gy). mRNA level was estimated 24 hours after irradiation. Two independent experiments were performed in quadruplicates (*p<0.05, **p < 0.01 and ***p < 0.001).

(B) Representative Western blot of HIF1α nuclear and cytoplasmic protein expression in HIF1α-knockdown TG1N cells (shHIF1α) and their respective control (shCt) treated or not with 50 µM YC1 (2 hrs) before DFO treatment (100 µM DFO; 2 hrs). Twenty-five µg of cytoplasmic and nuclear protein were analyzed by immunoblot. Quantification of nuclear HIF1α were normalized to lamin B1 signal. Three independent experiments were performed.

(C) RT-qPCR quantification of HIF1α mRNA levels in TG1N cells transduced with shCt or shHIF1α. Each condition was performed in duplicates (***p < 0.001).

**Supplementary Figure S6.**

**JMY pathway is involved in radiation-induced migration of GSCs.**

(A) JMY mRNA levels in TG16 cells at different times after irradiation (0.5 Gy). Each time-point was performed in quadruplicates (*p < 0.05, **p < 0.01 and ***p < 0.001).

(B) JMY fluorescence intensity in TG16 cells: unirradiated (-), irradiated (+, one hour post-irradiation at 0.5 Gy), pretreated with YC1 (50 µM) and irradiated (one hour post-irradiation at 0.5 Gy), and treated one hour with DFO (100 µM). Data were obtained from at least 28 cells per condition (**p < 0.01 and ***p < 0.001).

(C) RT-qPCR quantification of HIF1α mRNA levels in TG16 cells transduced with shCt or shHIF1α. Each condition was performed in duplicates (***p < 0.001).

(D) RT-qPCR quantification of JMY mRNA levels in control TG16 cells (shCt) and in HIF1a-deficient TG16 cells (shHIF1α). Each condition was performed in duplicates (**p < 0.01).

(E) RT-qPCR quantification of JMY mRNA levels in TG1N and TG16 cells transfected with siCt or siJMY. Each condition was performed in quadruplicates (***p < 0.001).

(F) Migration velocity of TG16 cells electroporated with a siRNA targeting JMY (siJMY) or a scrambled control siRNA 24 hours after irradiation (0.5 Gy). (n = 92 cells per condition; **p < 0.01).

(G) RT-qPCR quantification of JMY mRNA levels in TG1N cells transduced with shCt or shJMY. Each condition was performed in quadruplicate (***p < 0.001).

**Supplementary Figure S7.**

Quantification of mRNA coding for JMY (A), BNIP3 (B), GLUT1 (C) and VEGFA (D) mRNA levels in irradiated control (shCt) or deficient knockdown TG1N GSCs for HIF1α (shHIF1α) between 1 and 16 hours after irradiation (0.5 Gy). Significant differences between shCT and shHIF1α for a similar time point are noted ^#^p < 0.05, ^##^p < 0.01 and ^###^p < 0.001 whereas significant difference between 0h and others time post-irradiation for shCt or shHIF1α cells are noted *p < 0.05, **p < 0.01 and ***p < 0.001. Each time point was performed at least in triplicates from two independent experiments.

**SUPPLEMENTARY REFERENCES**

Gorelik, R. & Gautreau, A. Quantitative and unbiased analysis of directional persistence in cell migration. *Nat Protoc* **9**, 1931-1943, doi:10.1038/nprot.2014.131 (2014).

Munshi, A., Hobbs, M. & Meyn, R. E. Clonogenic cell survival assay. *Methods in molecular medicine* **110**, 21-28, doi:10.1385/1-59259-869-2:021 (2005).
