# Supplementary material for: The HIF1α/JMY pathway promotes glioblastoma stem-like cell invasiveness after irradiation
Source: Sci Rep. 2020 Oct 30;10:18742. doi: 10.1038/s41598-020-75300-5 (PMC7603339; doi:10.1038/s41598-020-75300-5)
Supplement: Supplementary file 2 — Supplementary Figures. [file 41598_2020_75300_MOESM2_ESM.pdf]

The HIF1α/JMY pathway promotes glioblastoma stem-like cell invasiveness after irradiation

Laurent R. Gauthier [1,*], Mahasen Saati [1], Hayet Bensalah-Pigeon [1], Karim Ben M'Barek [1,2], Oscar Gitton-Quent [1], Romane Bertrand [3], Didier Busso [1], Marc-André Mouthon [1] , Ada Collura [3], Marie-Pierre Junier [4], Hervé Chneiweiss [4], José R. Pineda [1,5], and François D. Boussin [1,*]

Supplementary Figure

A

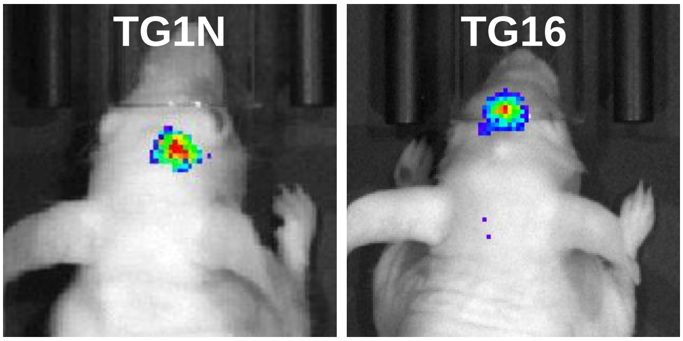

B

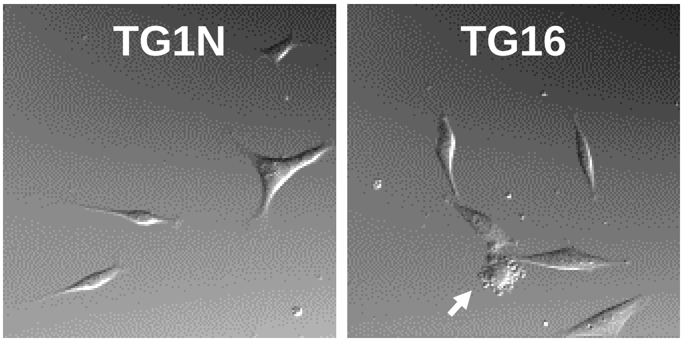

C

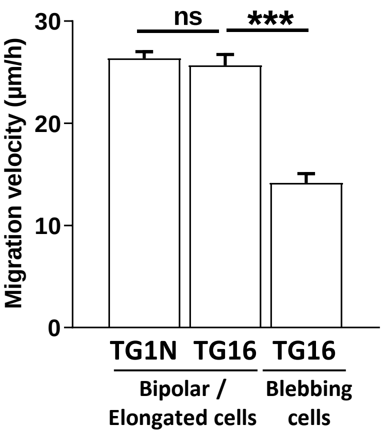

Supplementary Figure S1

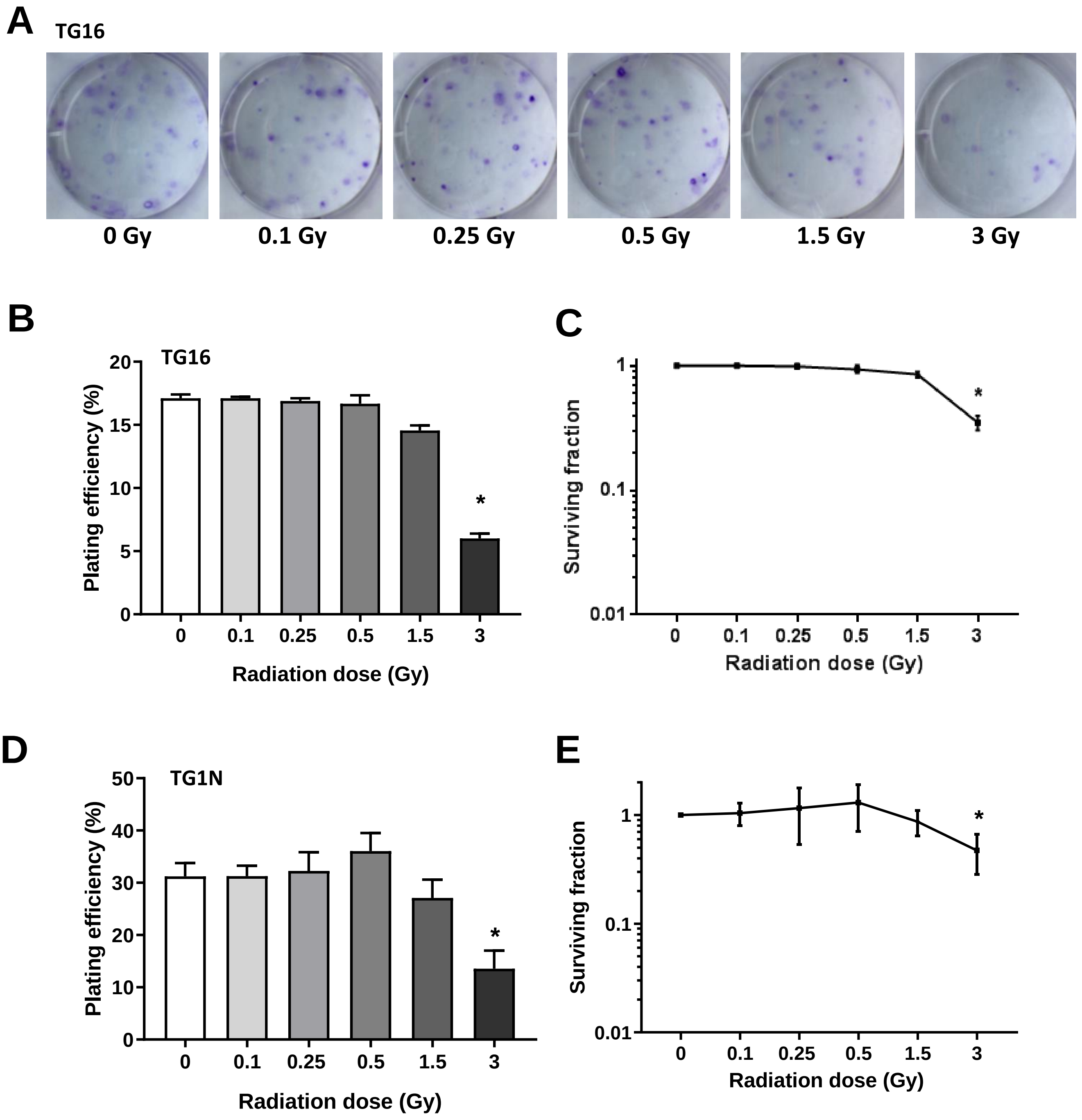

Supplementary Figure S2

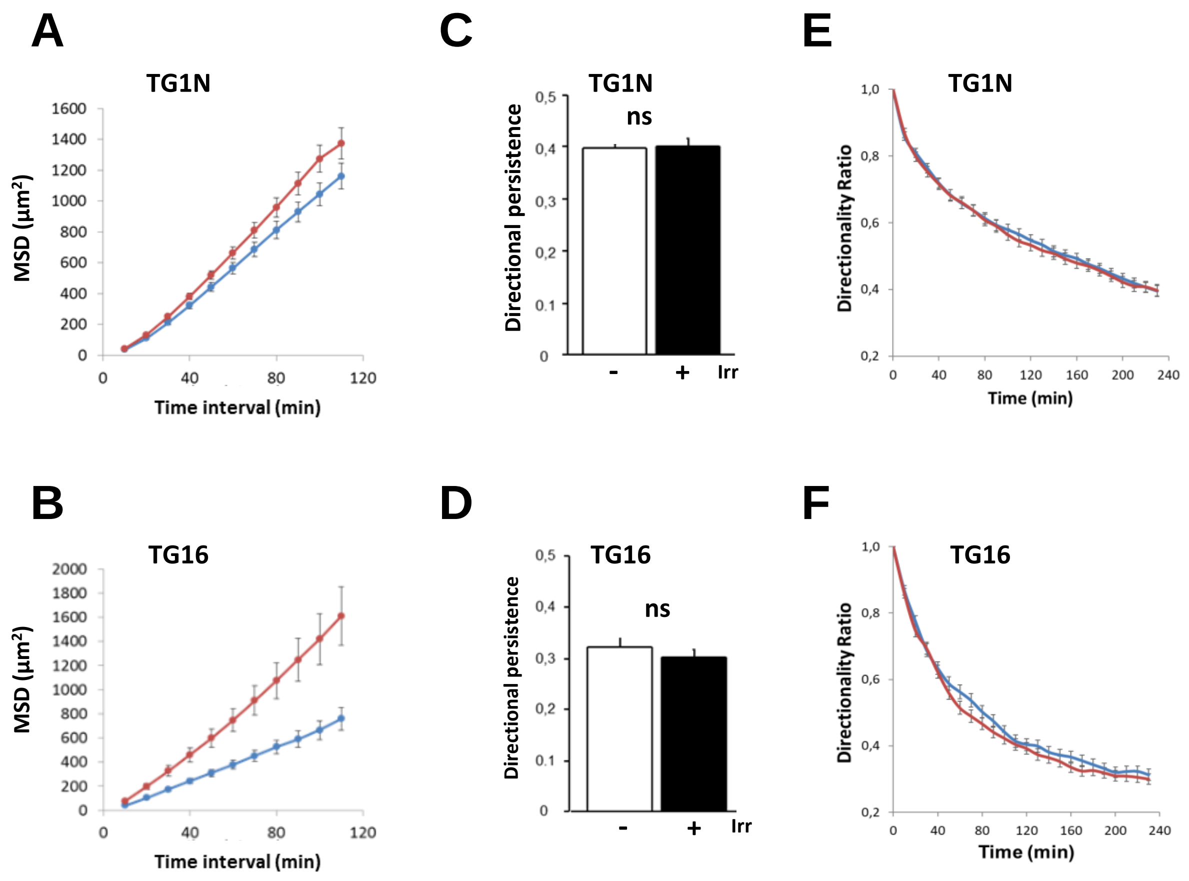

Supplementary Figure S3

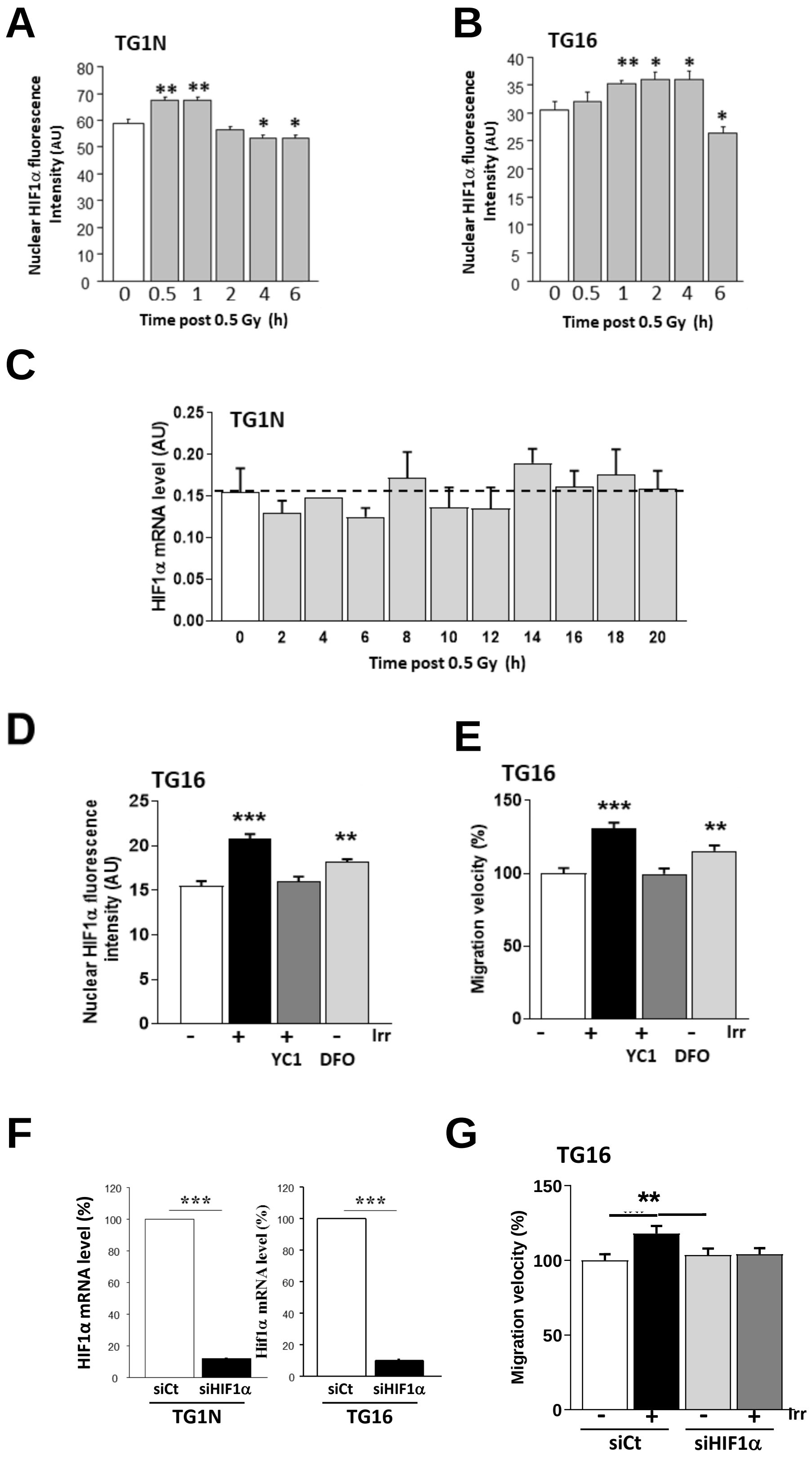

Supplementary Figure S4

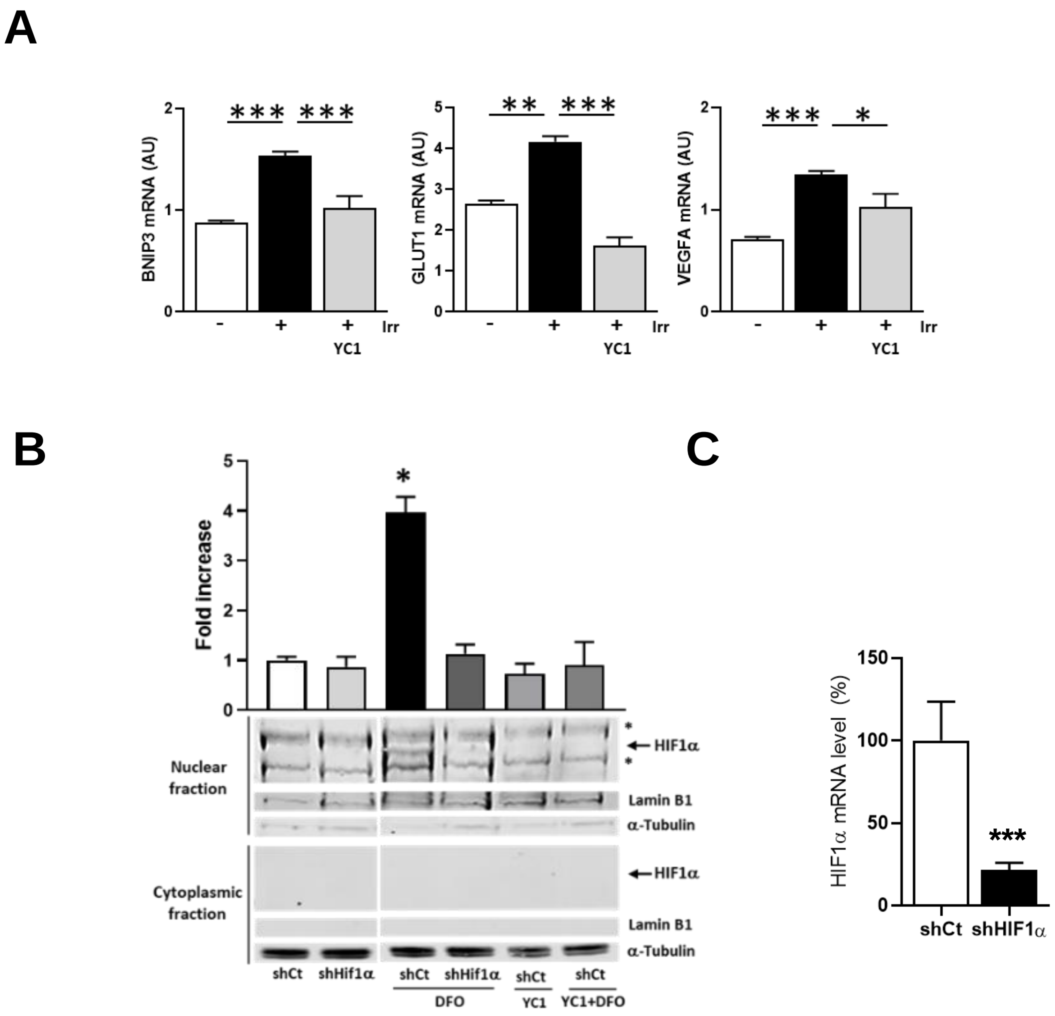

Supplementary Figure S5

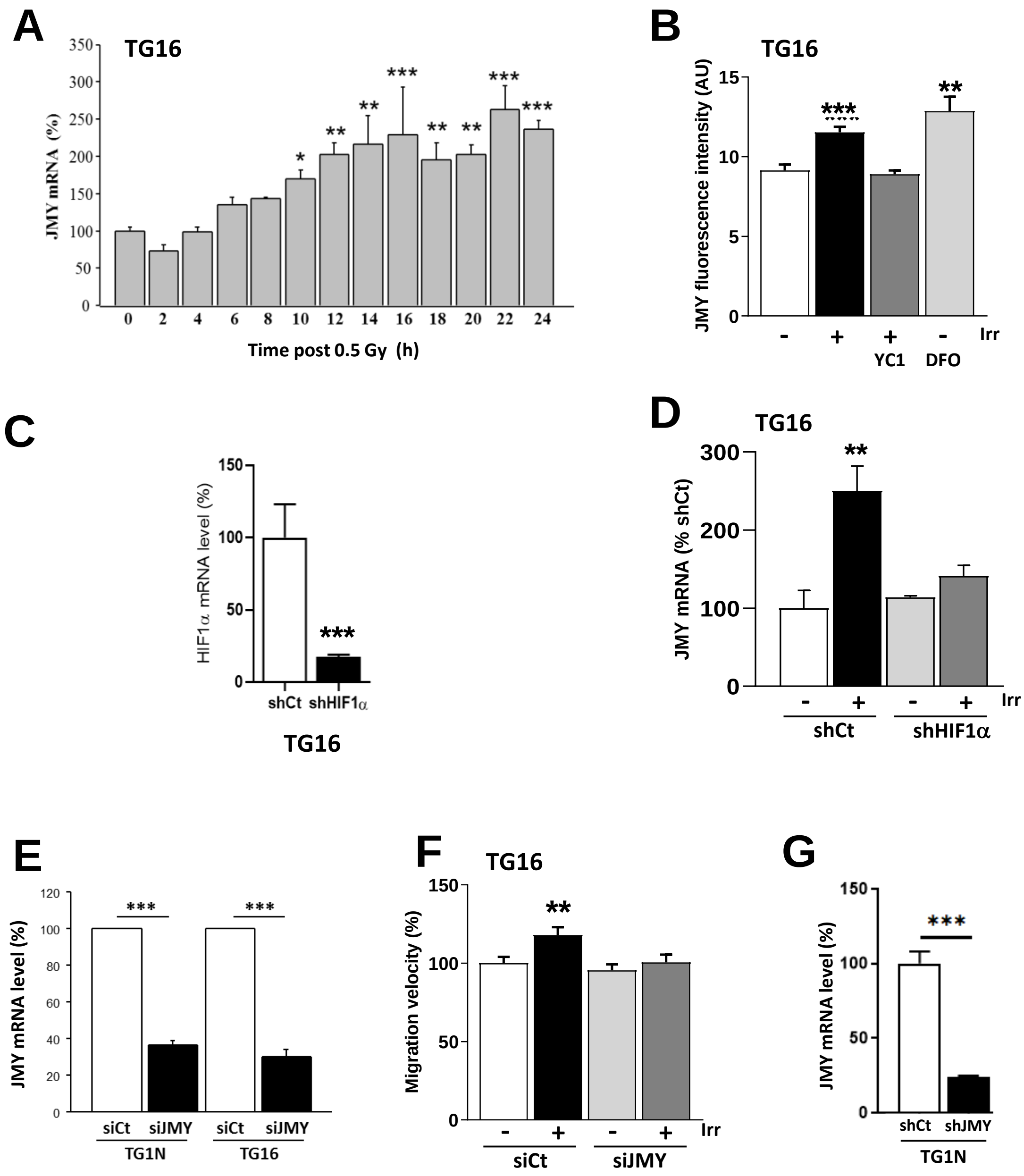

Supplementary Figure S6

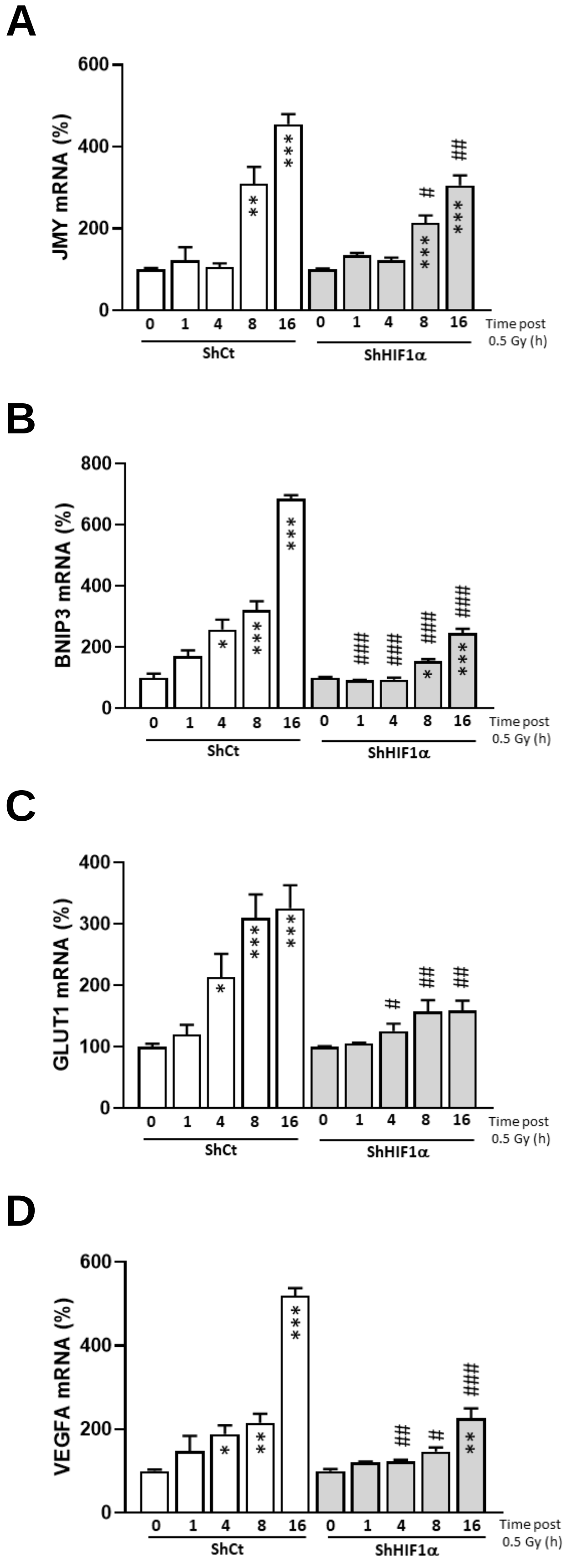

Supplementary Figure S7
